# Doping-Less Feedback Field-Effect Transistors

**DOI:** 10.3390/mi15030316

**Published:** 2024-02-24

**Authors:** Hakin Kim, Doohyeok Lim

**Affiliations:** 1 Department of Nano Electronic Convergence Engineering, Kyonggi University, Suwon 16227, Gyeonggi-do, Republic of Korea; 2 School of Electronic Engineering, Kyonggi University, Suwon 16227, Gyeonggi-do, Republic of Korea

**Keywords:** doping-less device, feedback field-effect transistor, charge plasma

## Abstract

In this study, we propose doping-less feedback field-effect transistors (DLFBFETs). Our DLFBFETs are 5 nm thick intrinsic semiconductor bodies with dual gates. Usually, DLFBFETs are virtually doped through charge plasma phenomena caused by the source, the drain, and the dual-gate electrodes as well as the gate biases. Our DLFBFETs can be fabricated through a simple process of creating contact between a metal and a silicon body without any doping processes. The voltages applied to both gates determine whether the DLFBFETs operate in diode or feedback field-effect transistor (FBFET) modes. In the FBFET mode, our DLFBFETs show good characteristics such as an on/off current ratio of ~10^4^ and steep switching characteristics (~1 mV/decade of current) that result from positive feedback phenomena without dopants.

## 1. Introduction

Under Moore’s law, metal–oxide–semiconductor field-effect transistors (MOSFETs) are scaled down, reducing the channel length of MOSFETs, which enhances the integration level and performance of the transistors. However, with the increase in the number of transistors in an integrated circuit or chip, the power dissipation problem has grown. To lower power consumption, the gate threshold voltage and leakage current must be kept low. Nevertheless, because the subthreshold swing (SS) of the existing MOSFETs is theoretically limited to 60 mV/dec at room temperature, MOSFETs eventually face limitations in exhibiting excellent switching characteristics. Even in FinFETs and gate-all-around structures which eliminate leakage paths, MOSFETs have limitations in reducing power consumption.

As alternatives, devices with an SS of less than 60 mV/dec have been proposed. These devices have surpassed the SS limitations by using new mechanisms, such as tunneling [1], impaction ionization [2,3], negative capacitance [4,5], and positive feedback loops [6,7,8], and have been studied as replacements for existing MOSFETs. Among these, feedback field-effect transistors (FBFETs) [6,7,8] are the most promising candidates for next-generation switching devices. FBFETs are field-effect transistors that utilize feedback phenomena and are operated by modulating potential barriers. This modulation is accompanied by a steep increase and decrease in the current. This allows FBFETs to operate at a low power in the on/off state by having a very low SS and a high on/off current ratio [6,7,8]. However, FBFETs exhibit more complex structures than other devices. Complex structures require sophisticated processing. In particular, during the doping process, random dopant fluctuation (RDF) hinders the stable performance of FBFETs and requires more elaborate doping processes. This issue becomes more pronounced when they are scaled down. Virtual doping, which concentrates carriers in ultrathin structures without chemical doping processes, is one of the solutions adopted to address these challenges [9]. Among these, the charge plasma concept is a type of virtual doping that utilizes energy band bending around metal junctions by contacting semiconductor bodies and other metals with different work functions to concentrate carriers. This charge plasma-based virtual doping has the advantage of achieving electrical properties similar to those of physically doped devices with only metal contact without needing separate elaborate processes. Additionally, it can bypass the RDF problem and enhance device performance.

Doping-less devices, which are virtually doped, include charge plasma diodes [10,11], bipolar charge plasma transistors [12,13,14], and doping-less junction-less transistors [15] exhibiting electrical characteristics similar to those of existing devices. Doping-less devices with an SS below 60 mV/dec, such as doping-less TFETs (TFETs) [16,17,18,19] and doping-less double-gate impact ionization MOSFETs (DLDGIMOS) [20], are also under study. Nevertheless, doping-less feedback field-effect transistors have not been reported.

In this study, we propose doping-less feedback field-effect transistors (DLFBFETs) based on the concept of charge plasma. Our devices feature a 5 nm thin body virtually doped with a source, a drain, and dual gates. Because they are virtually doped, our devices can be manufactured without several doping processes and are free from the RDF problems associated with the doping processes. Under certain gate biases, our devices exhibited a steep increase in current resulting from a positive feedback loop. Our devices demonstrated excellent switching characteristics through this positive feedback loop and band modulation, such as an on/off current ratio of ~10^4^ and steep switching characteristics of ~1 mV/dec.

## 2. Device Structure and Simulation

**Figure 1:**
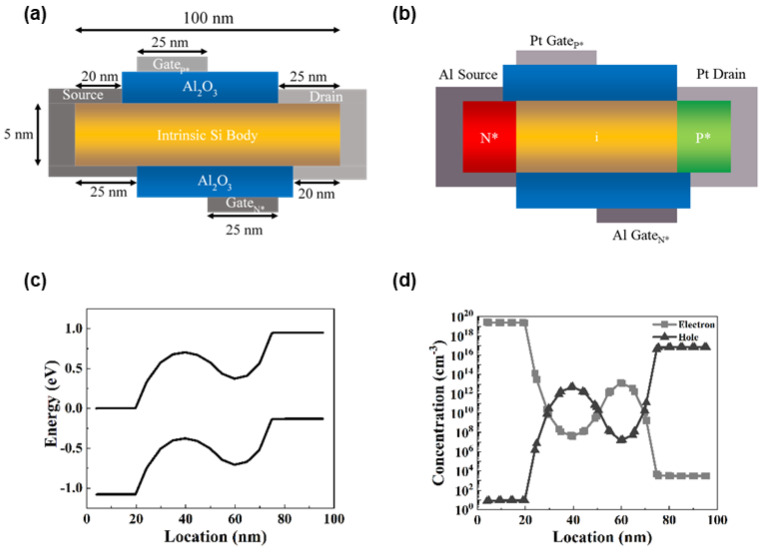
Schematic of DLFBFET. (**a**) Structure, (**b**) virtually doping type, (**c**) energy band diagram, and (**d**) carrier concentration under no bias.

Figure 1a shows the structure of a DLFBFET. The width of the silicon body is 1 μm, the thickness is 5 nm, and the length is 100 nm. The lengths of the source, drain, and both gates are all 25 nm, with a 5 nm gap at the top of the source and at the bottom of the drain. The oxide layers consist of Al_2_O_3_ with a thickness of 5 nm and a length of 55 nm.

Figure 1b shows that the intrinsic silicon body (n = p = 1 × 10^10^ cm^−3^) is virtually doped without external bias through the charge plasma phenomenon. The source and Gate_N*_ electrodes are composed of aluminum with a work function of 4.17 eV. Consequently, the aluminum electrodes exhibit a higher electric potential than the intrinsic silicon body, whose work function is 4.61 eV. Because of the charge plasma phenomenon between the aluminum electrodes and the silicon body, electrons accumulate in the region around the source. This implies that these regions are virtually doped as N*-type regions. By contrast, the drain and Gate_P*_ electrodes consist of platinum with a work function of 5.12 eV, and the electric potential of the platinum electrodes is lower than that of the Si body. Holes accumulate in the region around the drain owing to the charge plasma phenomenon. As a result, the intrinsic Si body becomes similar to that of a P-i-N diode due to the charge plasma phenomena.

Figure 1c,d show the variations in the electric characteristics through the charge plasma phenomena of the intrinsic silicon body in the off state. Figure 1c shows the energy band diagram of a silicon body under no external bias. The energy band is lower around the source and above Gate_N*_ (virtually doped regions of N*-type) and higher around the drain and below Gate_P*_ (virtually doped regions of P*-type). The carrier concentration of a virtually doped body is shown in Figure 1d. The electron concentration around the source is approximately 2.5 × 10^19^ cm^−3^, and, above Gate_N*_, it is approximately 1.5 × 10^13^ cm^−3^. The hole concentration around the drain is approximately 7 × 10^16^ cm^−3^, and, under Gate_P*_, it is approximately 5 × 10^12^ cm^−3^.

We conducted two-dimensional structure simulations using a commercial device simulator (Silvaco ATLAS, Version 5.2.17. R) to investigate the DLFBFET. Because our DLFBFET has an N*–P*–N*–P* structure, we employed a BJT model to analyze the P-N-P and N-P-N bipolar junctions. Additionally, we utilized the CVT mobility, Klassen mobility, and Shockley−Read−Hall recombination bandgap narrowing models. The default parameters for these models were used in the simulation.

## 3. Results and Discussion

**Figure 2:**
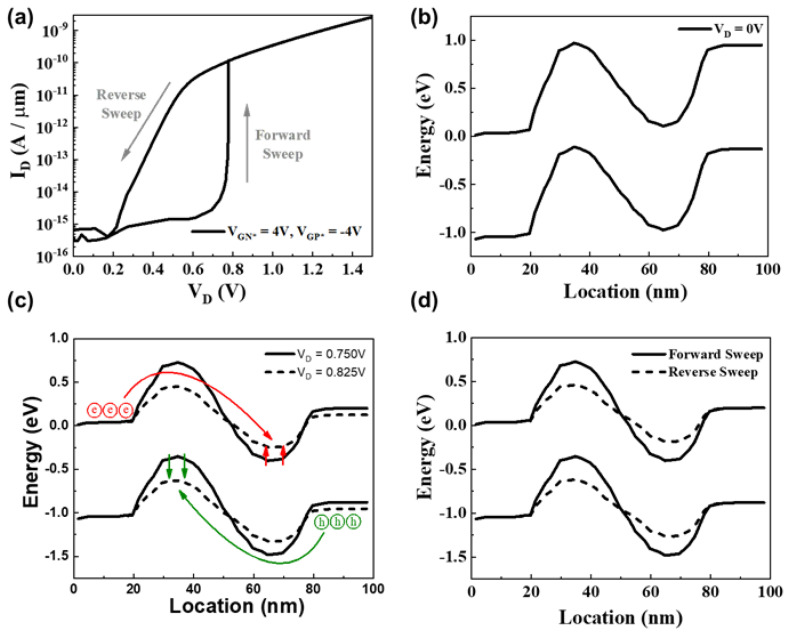
(**a**) IV characteristic graph at V_GN*_ = 4 V and V_GP*_ = −4 V. Energy band diagrams (**b**) under no drain bias, (**c**) before (V_D_ = 0.75 V) and after (V_D_ = 0.825 V) the latch phenomenon, and (**d**) of the hysteresis phenomenon at V_D_ = 0.75 V.

Figure 2a presents an IV characteristic graph with gate biases V_GN*_= −4 V and V_GP*_ = 4 V. At V_D_ = 0.77 V, an abrupt increase in the current (latch-up phenomenon) was observed, and the steepest inverse slope of the logarithmic I_D_ vs. V_D_ curve was approximately 1 mV/dec. Compared to conventional diodes with a theoretical limit of 60 mV/dec for d(logI_D_)/dV_D_, DLFBFETs exhibit steep switching characteristics. During the reverse sweep of V_D_ from 1.5 V back to 0 V, a hysteresis phenomenon in the current, indicating band modulation, was evident. The on/off current ratio at V_D_ = 0.6 V was approximately 2.24 × 10^4^.

The energy band diagram for V_D_ = 0 V is shown in Figure 2b. Through virtual doping via gate biases, hole and electron barriers are formed in the channel, resulting in our devices having an N*-P*-N*-P* structure. In addition, owing to virtual doping by gate biases, our device does not suffer from the reliability issues caused by single-charged defects. Figure 2c compares the energy band diagrams before the latch phenomenon (V_D_ = 0.75 V) and after (V_D_ = 0.825 V). Notably, the potential barriers after the latch-up phenomenon are lower than those before the latch phenomenon in channel N*—a virtually doped channel region located over Gate_N*_. This phenomenon suggests the occurrence of band modulation resulting from a positive feedback loop.

As V_D_ increases, the hole barrier height gradually decreases, and the holes in the drain are injected into the channel. The injected holes accumulate in the channel P*—virtually doped region located below Gate_P*_—and eventually flow into the source as majority carriers. The hole accumulation in channel P* increases its electric potential, thereby reducing the electron barrier height. The reduction in the electron barrier height allows the electrons in the source to be injected into the channel. Similar to the hole injection mechanism, injected electrons accumulate in channel N* and decrease the electric potential of channel N*. The lowered hole–barrier height allows more holes in the drain to be injected into the channel. This process is repeated to form a positive feedback loop. The band modulation through this positive feedback loop is accompanied by an abrupt turn-on, which corresponds to a latch-up phenomenon.

Figure 2d illustrates the energy band diagram of the hysteresis phenomenon. Owing to the band modulation caused by the positive feedback phenomenon, the potential barriers for the electrons and holes during the reverse sweep are smaller than those during the forward sweep, even though V_D_ is the same (0.75 V). Once turned on through the feedback phenomenon, the DLFBFETs exhibit low potential barrier heights. As the drain voltage is swept downward, the hole barrier height increases again, and fewer holes from the drain are injected into the channel. As the number of holes injected from the drain decreases, the positive feedback loop is suppressed. Consequently, the carrier barriers increase again, and I_D_ decreases. In the reverse sweep, this band modulation occurs gradually, resulting in a lower barrier height than that in the forward sweep. The lower barrier height formed by the accumulated charge carriers in the potential wells results in a greater current. The forward/reverse current ratio at V_D_ = 0.75 V is approximately 8.24 × 10^3^. Owing to these electrical characteristics, our DLFBFETs show promise as switching devices.

**Figure 3:**
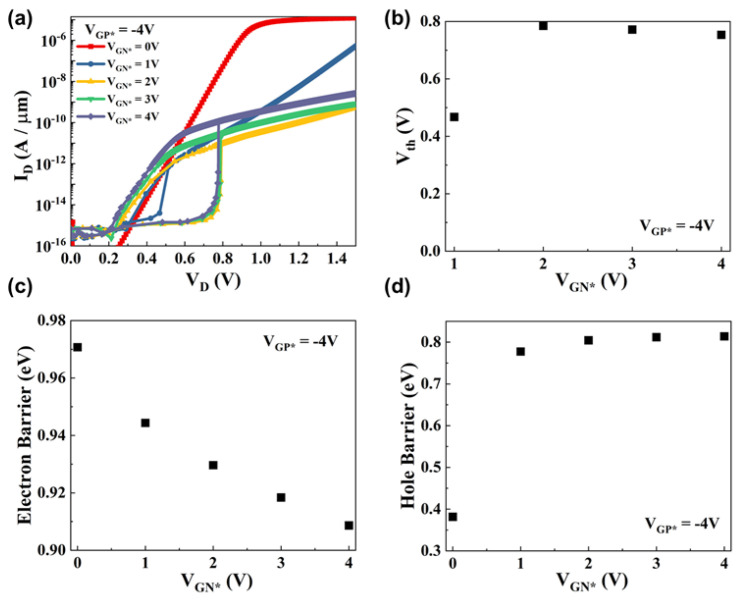
Electrical characteristics of DLFBFETs under various V_GN*_ conditions. (**a**) IV output characteristic curves, (**b**) threshold voltages, (**c**) electron barriers, and (**d**) hole barriers.

Next, we discuss the latch-up phenomenon under various gate biases. For V_GP*_ fixed at −4 V, IV output characteristic graphs under various values of V_GN*_ are plotted in Figure 3a, and the threshold voltages are shown in Figure 3b. When V_GN*_ = 0 V, the latch-up phenomenon does not occur. This indicates that a positive feedback loop has not been generated. Without a positive feedback loop and band modulation, our DLFBFETs operate like a P-i-N diode. When V_GN*_ = 1 V, a relatively low threshold voltage and the latch-up phenomenon are observed. Under these conditions, the holes injected into the channel accumulate in channel P* and, shortly thereafter, cause a positive feedback phenomenon. Because the potential barrier created by V_GN*_ provides a low gain for the positive feedback loop, the switching characteristics are not as steep as those of a higher V_GN*_. When V_GN*_ is between 2 and 4 V, a noticeable latch-up phenomenon appears. Under these conditions, charge carrier accumulation, positive feedback loops, and band modulation are evident, and our DLFBFETs exhibit good switching characteristics. Moreover, the threshold voltage tends to decrease slightly as V_GN*_ increases. In our research, we analyzed these electrical characteristics based on the carrier barrier heights. Electron barrier heights were calculated by subtracting the average conduction band energy in the source region from the peak conduction band energy in the channel region below Gate_P*_. And hole barrier heights were calculated by subtracting the minimum valence band energy in the channel region above Gate_N*_ from the average valence band energy in the drain region. Figure 3c,d show the electron and hole barrier heights according to V_GN*_, respectively. As V_GN*_ increases from 0 to 4 V, the hole barrier height increases, and the electron barrier formed by the Gate_P*_ bias decreases slightly. When V_GN*_ = 0 V, the electron barrier is formed at about 0.97 eV and the hole barrier at about 0.38 eV. Under this condition, holes injected from the drain into the channel, unlike in the other graphs, easily cross the hole barrier, and the electric potential of channel P* increases with the drain voltage, as if the hole barrier is suppressed and not present. Therefore, the I–V output characteristic graph at V_GN*_ = 0 V does not show a latch-up phenomenon, and the DLFBFETs operate like a simple P-i-N diode.

When V_GN*_ = 1 V, the electron barrier height is approximately 0.94 eV, and the hole barrier height is around 0.78 eV. Under these conditions, a low V_D_ value triggers a positive feedback loop with a low gain, owing to the insufficient height of the hole barrier, which hinders the generation of a positive feedback loop with a high gain. A small on/off current ratio of ~10^2^ at V_GN*_ = 1 V is shown in Figure 3a, compared to the on/off current ratio of ~10^4^ at V_GN*_ = 4 V. If the hole–barrier height increases further with increasing V_GN*_ values, both the on/off current ratio and the threshold voltage also increase. The generation of a positive feedback loop with a high gain results in stable and good switching characteristics when V_GN*_ is between 2 and 4 V. However, as V_GN*_ increases, the hole barrier height slightly increases, whereas the electron barrier decreases. This reduction in the electron barrier creates a positive feedback loop at lower drain voltages. Consequently, the threshold voltages slightly decrease when V_GN*_ is between 2 and 4 V.

**Figure 4:**
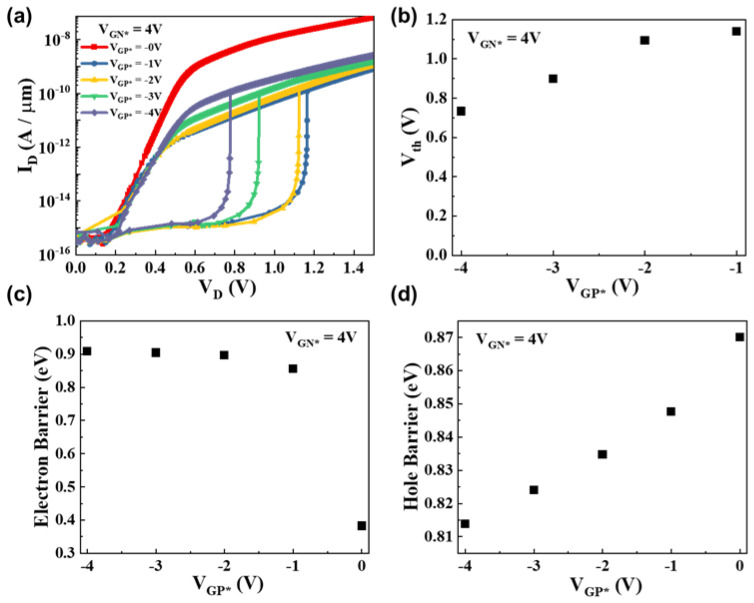
Electrical characteristics of DLFBFETs under various V_GP*_ conditions. (**a**) IV output characteristic curves, (**b**) threshold voltages, (**c**) electron barriers, and (**d**) hole barriers.

In our experiment, we also analyzed the electrical properties under various V_GP*_ conditions when V_GN*_ was fixed. Figure 4 illustrates the electrical characteristics of the DLFBFETs under different V_GP*_ conditions. For V_GN*_ fixed at 4 V, the I–V output characteristic graphs under various V_GP*_ conditions are presented in Figure 4a, and the threshold voltages are depicted in Figure 4b. At V_GP*_ = 0 V, there is no latch-up phenomenon, and our DLFBFETs operate like P-i-N diodes. This suggests that positive feedback and band modulation have not occurred. When V_GP*_ is between −1 and −4 V, noticeable latch-up phenomena appear, and the threshold voltage tends to decrease. Under these conditions, positive feedback loops with high gains are formed, and band modulations occur. In particular, in the case of V_GP*_ = −1 V, compared to the case of V_GN*_ = 1 V in Figure 3a, latch-up phenomena appear at a relatively high drain voltage. Figure 4c,d show the carrier barrier heights corresponding to different V_GP*_ values. As the fixed V_GP*_ values increase from −4 to −1 V, the threshold voltages shift from 0.73 to 1.14 V, as shown in Figure 4b. When the fixed V_GP*_ is varied from 0 to −4 V, the electron barrier heights increase, and the hole barriers formed by the V_GN*_ biases slightly decrease. This reduces the threshold voltage values depending on the decrease in the fixed V_GP*_ values.

## 4. Conclusions

In this study, we demonstrated DLFBFETs that utilize charge plasma phenomena and dual-gate biases. With the electrical properties induced by virtual doping, our DLFBFETs exhibited positive feedback phenomena inside the channel and demonstrated excellent switching properties such as steep switching characteristics of ~1 mV/dec and an I_on_/I_off_ ratio of approximately 10^4^. Because our DLFBFETs were manufactured without undergoing various processes, including doping, we could avoid potential issues that may arise in CMOS processes, such as random dopant fluctuation (RDF) problems and the challenges associated with scaling down. This makes the fabrication more straightforward. Compared to traditional FBFETs, which require complex structures and processes, our DLFBFETs combine excellent switching characteristics with ease of processing. Owing to their favorable switching properties and simplified fabrication process, our DLFBFETs present a promising option for next-generation switching devices.

## Data Availability

All data generated or analyzed during this study are included in this published article. The datasets used and/or analyzed in the current study are available from the corresponding author upon reasonable request.
